# Changes in Uterine Cancer Incidence Rates in Egypt

**DOI:** 10.1155/2018/3632067

**Published:** 2018-06-14

**Authors:** Saad Alshahrani, Amr S. Soliman, Ahmed Hablas, Mohamed Ramadan, Jane L. Meza, Steven Remmenga, Ibrahim A. Seifeldein, Robert M. Chamberlain

**Affiliations:** ^1^Department of Epidemiology, College of Public Health, University of Nebraska Medical Center, Omaha, NE, USA; ^2^Department of Community Health and Social Medicine, School of Medicine, City University of New York, New York, NY, USA; ^3^Gharbiah Cancer Society and Gharbiah Cancer Registry, Tanta 31512, Egypt; ^4^Department of Biostatistics, College of Public Health, University of Nebraska Medical Center, Omaha, NE, USA; ^5^Department of Obstetrics and Gynecology, University of Nebraska Medical Center, Omaha, NE, USA; ^6^Department of Epidemiology, University of Texas, M.D. Anderson Cancer Center, Houston, TX, USA

## Abstract

**Background:**

Uterine cancer is one of the top-ranking cancers in women with wide international variations in incidence rates. Developed countries have higher incidence rates than the developing countries. Egypt has significantly lower incidence of uterine cancer than other countries in the Middle East. This study aimed at verifying the incidence rate of uterine cancer and characterizing the demographic and clinical profiles of patients residing in the Gharbiah province in the Nile delta region of Egypt.

**Methods:**

Data from 660 uterine cancer patients diagnosed during the period of 1999 to 2010 were abstracted from the Gharbiah Cancer Registry, the only population-based registry in Egypt. The data included age, marital status, number of children, residence, smoking, occupation, date and basis of diagnosis, tumor topography, morphology, stage and grade, and treatment. Crude rate, age-standardized rate (ASR), and age-specific rate were calculated and associated with demographic and clinical characteristics of patients.

**Results:**

The study confirmed the low ASR of uterine cancer in Egypt, (4.1 per 100,000 (95% CI: 3.8–4.4)). The incidence rate increased significantly over the 12-year period. The crude rate (CR) was 1.95, 95% CI (1.64–2.25) in 1999–2002; 2.9, 95% CI (2.5–3.2) in 2003–2006; and 3.5, 95% CI (3.1–3.9) in 2007–2010. The rate ratio was 1.5, 95% CI (1.2–1.8) in 2003–2006 and 1.8, 95% CI (1.5–2.2) in 2007–2010 compared to 1999–2002. The majority of patients (83%) were postmenopausal with the highest age-specific rate in the 60–69-year age group (22.07 per 100,000 (95% CI: 19.3–25.2). The majority of patients were diagnosed at early stages (60% localized and 5% regional), had adenocarcinoma (68%), and resided in urban areas (54%).

**Conclusions:**

The study confirmed the low incidence rate of uterine cancer in the Gharbiah province of Egypt and significant increase in incidence in recent years. Future studies should focus on verifying the possible effect of hysterectomy on lowering the incidence, the factors related to the changes in rates between rural and urban areas, and the possible impact of nutritional and epidemiologic transitions on the increasing rates.

## 1. Introduction

Globally, uterine cancer is one of the top-ranking cancers that affect women [1, 2]. Endometrial cancer arises from the inner layer (endometrium) of the uterus, the origin of approximately 90% of uterine cancers, followed by uterine sarcoma that arises from the outer layer (myometrium) (8%) and less frequent types of cancer (2%).

According to the latest figures of the International Agency for Research on Cancer (Globocan 2012), corpus uteri cancer ranks as the fifth most common women's cancer worldwide after breast, colorectal, cervix uteri, and lung cancers [1, 3]. Globocan reported the aggregate age-standardized incidence and mortality rates of corpus uteri cancer as 8.2 and 1.8 per 100,000, respectively [1]. Uterine cancer has a different incidence distribution in different regions of the world. It is the most common gynecologic malignancy in developed countries and the second most common gynecologic malignancy in developing countries after cervical cancer [1, 4]. North America and Central and Eastern Europe have the highest incidence of corpus uteri cancer in the world [1, 3]. In contrast, some regions in Africa show the lowest incidence rate in the world [5–7].

The variation in uterine cancer incidence rates across the world can be explained by differences in exposure to risk factors and different levels of health care in the different regions [8].

Based on Globocan, corpus uteri cancer is ranked as the tenth most common cancer among women in Egypt. According to the Middle East Cancer Consortium (MECC) Report of 2006, the incidence rate of uterine cancer in Egypt (3.5/100,000) is the lowest compared to other countries in the Middle East such as Israeli Jews (13.8/100,000), Cypriots (11.8/100,000), Israeli Arabs (8.7/100,000), and Jordanians (5.8/100,000) [9]. In addition, the low stage at diagnosis of uterine cancer is high compared to other female cancers in Egypt [9].

This study incorporated the most recent data collected by the Egyptian registry after the published report on the data of 1999–2002. We aimed at verifying the suspected increasing incidence rate of uterine cancer and characterizing the demographic and clinical profiles of patients residing in the Gharbiah province of Egypt during the period of 1999–2010.

## 2. Methods

### 2.1. The Study Population

The study included all primary uterine cancers that were diagnosed in the Gharbiah province and collected by the Gharbiah Population-Based Cancer Registry (GPCR) from January 1999 through December 2010. The registry was founded in 1998 as a part of the Middle East Cancer Consortium. The Gharbiah Cancer Society is the home of GPCR in Tanta, which is the capital city of the Gharbiah province in the center of the Nile delta region. The registry uses the International Agency for Research on Cancer's (IARC) software CanReg4, and the data were actively collected from all hospitals and pathologic labs of the Gharbiah province. In addition, patients seeking medical care outside the Gharbiah province are tracked, and their data are abstracted and included in the registry.

The Gharbiah province is about 100 km north of Cairo in the Nile delta region. It is the tenth largest province in Egypt with a total area of 1,948 km^2^. It consists of 8 districts. These include El-Mahalla El-Kubra, Kafr El-Zayat, Samannoud, Tanta, Zifta, El-Santa, Kotoor, and Bassyoun. The total population of the Gharbiah province according to the Egyptian census of 2006 was 4,011,320 individuals that represent 5.5% of Egypt's total population. The study was approved by the Institutional Review Board (IRB) at University of Nebraska Medical Center (UNMC) and Gharbiah Cancer Center Ethics Committee.

### 2.2. Data Sources

Cases were coded in the registry based on the third edition of the International Classification of Diseases for oncology (ICD-1O-3) coding systems. Cases included C54.0–C54.9 (corpus uteri cancer) and C55.9 (uterus, NOS cancer). There were 660 cases of uterine cancer reported during the period of 1999–2010 in the Gharbiah province. The demographic and clinical characteristics of the patients that were abstracted included registration number of the patients, age, marital status, number of children, place of residence, smoking status, occupational status, date and basis of diagnosis, tumor topography, tumor morphology, tumor stage, tumor grade, and treatment.

The population data of the latest Egyptian census of 2006 were obtained from the data of the Central Agency for Public Mobilization and Statistics (CAPMAS) [10].

### 2.3. Data Management and Statistical Analysis

The histopathologic types of the tumors were grouped into 4 types based on the histopathologic grouping of the International Agency for Research on Cancer (WHO) used in Cancer Incidence in Five Continents Volume IX (CI5) [11].

The uterine cancer morphology codes were grouped into 4 main subgroups. These are carcinoma (8010–8574 and 8576), sarcoma (8800–8811, 8830, 8840–8921, 8990–8991, 9040–9044, 9120–9133, 9150, and 9540–9581), and other specified malignant neoplasm and unspecified malignant neoplasm (8000–8005). Carcinoma was divided into adenocarcinoma (8140–8141, 8190–8211, 8230–8231, 8260–8263, 8310, 8380, 8382–8384, 8430, 8440–8490, 8510, 8560, 8570–8574, and 8576) and other specified carcinoma and unspecified carcinoma (8010–8035).

Tumor grades were classified into 4 grades: Grade I, for well differentiated, Grade II, for moderately differentiated, Grade III, for poorly differentiated, and Grade IV, for undifferentiated anaplastic tumors, and lastly, the Unspecified Grade. Tumor stages were grouped into 4 stages: local, regional, distal, and unstaged.

The incidence rate (IR) was calculated by dividing the number of events (E) by the total number of population at risk (P) per 100,000 women. The 95% confidence interval (CI) was calculated using the following formula: CI = IR ± 1.96 × IR/√E, and the 95% CI of rate ratio was calculated by using CI = exp (In (IR1/IR2)) ± 1.96 × √(1/E1 + 1/E2). The Egyptian census of 2006 was used to determine the number of women at risk.

The incidence rate of uterine cancer was calculated for both pre- and postmenopausal women. The age of 50 years was used to determine the menopausal status. Due to the low number of cases, the incidence rates were compared over the 3 periods: 1999 to 2002, 2003 to 2006, and 2007 to 2010. The crude incidence rate was used to compare the incidence of uterine cancer over the entire time frame of the study to the initial incidence in the period of 1999 to 2002. The person-years at risk was calculated over the 12-year period based on the total population of Gharbiah women in the census of 2006. The world standard population (WHO 2000) was used to calculate the age-standardized rate.

Chi-square tests and *t*-tests were used to test the associations of categorical and continuous variables, respectively. For all the analyses, a two-sided *p* value of ≤0.05 was considered statistically significant. SAS version 9.4 (SAS Institute Inc., Cary, NC) was used in all statistical analysis.

## 3. Result

There were 660 women diagnosed with primary uterine cancer in the Gharbiah province during the period of January 1999 through December 2010.  Table 1 shows the distribution of demographic and clinical characteristics of the patients included in this study. About 83% of patients were postmenopausal, and 54% lived in urban areas. Most patients (93%) were diagnosed based on the histopathologic diagnosis. However, less than 5% of patients were diagnosed with uterine cancer based on death records only. About 91% of the patients had corpus uteri cancer, and 9% had the diagnosis of “uterus NOS” (not otherwise specified). Adenocarcinoma and other carcinomas comprised 80% of the patients. Sarcoma represented 11% of the tumors, and 9% of all tumors were “unspecified malignant neoplasms.” Surgery alone was the most offered treatment in 48% of treated patients, followed by surgery and adjuvant radiotherapy in 19% of patients who had treatment information reported. Treatment data showed that 23% of the patients either did not receive any kind of treatment because they were not fit for the treatment or treatment was not available or affordable. In addition, 27% of all patients' treatment data were missing because the treatment data were not available in the registry from the beginning, which resulted in “missing” in all those patients.

The overall crude incidence rate of uterine cancer in Gharbiah was 2.8 per 100,000 with 95% CI (2.6–3.0). The age-standardized incidence rate was 4.1 per 100,000 with 95% CI (3.8–4.4). Unsurprisingly, the crude incidence rate of uterine cancer was 15.9 per 100,000 in postmenopausal women with 95% CI (14.6–17.3), whereas the premenopausal women were only 0.57 per 100,000 and 95% CI (0.46–0.67). The crude rate ratio was 28.1 and 95% CI (23.0–34.4). Therefore, postmenopausal women had 28.1 times the odds to develop uterine cancer compared to premenopausal women.

Table 2 shows the age-specific incidence rate of uterine cancer. Descriptively, the age-specific rate peaked at 22 per 100,000 and 95% CI (19.28–25.16) in the age group of 60–69 years, while the age-specific rate was lower in subsequent age groups.

The crude incidence rate of uterine cancer in the Gharbiah province over the period of 1999 through 2002 was 1.95 per 100,000 and 95% CI (1.6–2.3), and for the period of 2003 through 2006, it was 2.9 per 100,000 and 95% CI (2.5–3.2). By 2007 to 2010, the crude rate had increased to 3.5 per 100,000 and 95% CI (3.1–3.9). The crude rate was higher in 2003–2006 and 2007–2010 compared to 1999–2002. The crude rate of uterine cancer in the period of 2007 to 2010 was 1.8 times the rate in the period of 1999 to 2002.  Table 3 shows the uterine cancer crude rate and the increment in the rate ratio over the time with the significant 95% CI.

Corpus uteri cancer comprises the majority of uterine cancers in both pre- and postmenopausal women with proportions of 83% and 93%, respectively (Table 4). However, the NOS cancer has a higher proportion in premenopausal women (17%) than (7%) in postmenopausal women (*p* < 0.001).

Carcinomas were the most common type in pre- and postmenopausal women, 61% and 84%, respectively. The tumor grades have significant association with the menopausal status (*p* < 0.001). About 10% and 15% of pre- and postmenopausal, respectively, were in well-differentiated tumor grade. Moderately differentiated tumor comprised 38% of premenopausal and 47% of postmenopausal patients. Unspecified tumor grade had the highest percentage of premenopausal women (41%), whereas only 20% of postmenopausal women had unspecified tumor grade. Poorly and anaplastic tumor grade comprised 11% and 18% of pre- and postmenopausal patients, respectively.

Although the nulliparity was relatively high in both premenopausal and postmenopausal (52% and 48%, resp.), the parity levels did not show a significant difference by menopausal status (*p*=0.35). Moreover, treatment, residential status, and employment status did not have any significant association with the menopausal status (Table 4).

Table 5 displays the association of histopathologic types with different clinical characteristics of uterine cancer. About half the carcinoma patients were diagnosed with moderate tumor grade. Histopathologic types of cancer had significant differences across the levels of tumor grades, 59% of adenocarcinoma were moderately differentiated, 53% of other carcinoma were unspecified tumor grade, and 32% of sarcomas were diagnosed with poor and anaplastic tumor grade (*p* < 0.001). All sarcomas (100%) were diagnosed as corpus uteri, whereas 75% of other and unspecified malignancies were diagnosed as NOS uterine cancers. The highest proportions of all histopathologic types that were treated with surgery only were sarcomas (63%) followed by adenocarcinomas (48%). Histopathologic types of cancer had significant differences in relation to the given treatment (*p* < 0.001).

## 4. Discussion

This study revealed 3 interesting findings. First, uterine cancer ASR in this population was relatively low during the entire study period of 1999–2010; however, the crude and ASRs increased significantly by 2010. Second, the peak age-specific incidence rate of uterine cancer was in the age group of 60–69 years. Third, adenocarcinoma and other carcinomas comprised about 80% of all uterine cancers in this population.

There are a limited number of studies examining ASR of uterine cancer in Egypt. The first population-based study was published by our group, for data from 1999 to 2001, in the monograph of the Middle East Cancer Consortium (MECC) [9]. The MECC report included the period 1999–2001 and showed an ASR of 3.5 per 100,000 in this population in Egypt compared to ASR of 5.8 per 100,000 for Jordan, ASR of 8.7 per 100,000 for Israeli Arabs, ASR of 13.8 per 100,000 for Israeli Jews, ASR of 11.8 per 100,000 for Cyprus, and ASR of 17.6 per 100,000 for SEER, USA [9]. A more recent study by members of our group showed a crude incidence rate of uterine cancer of 1.91 per 100,000 for the same population for the period of 1999 to 2002 [12]. However, based on the Cancer Incidence in Five Continents Vol. X [3], other Arab countries have higher ASRs than the Egyptian rates. For example, Saudi Arabia, Kuwait, and Qatar had ASRs of 4.7, 6.7, and 8.4 per 100,000, for the 3 countries, respectively [3]. Rates reported from developed countries in Europe and North America were significantly higher than the Egyptian rates (19.5, 17.0, and 13.9 per 100,000 for the US, France, and the U.K., resp.) [2]. In addition, the world uterine cancer ASR as reported in Globocan 2012 was 8.2 per 100,000 [2]. Therefore, the results of this study clearly show that the Egyptian ASR of uterine cancer is considered one of the lowest uterine cancer incidence rates, globally.

Regarding the trend of increasing uterine cancer crude rates in Egypt in this study, other studies showed increasing trends of uterine cancer incidence in some European countries. For example, the incidence of uterine cancer increased significantly in the period of 1988 to 2008 in Norway, Ireland, the United Kingdom, and the Netherlands [13]. Some Asian countries also showed continuous increase in uterine cancer incidence rates. For example, 5 of the 6 population-based cancer registries in India showed an increase in uterine cancer trend over the period of 1982 to 2003 [14]. In addition, the increment in the uterine cancer trend was noticed also in Japan over the period of 1983 to 2005 [15]. In contrast, the rates declined in Austria, Sweden, and Germany [13]. In the US, the trend showed declining rates for 3 decades until the 1990, stability in the 1900s, then slight increase after 2007 [16].

The significant variations in ASRs across different countries could be explained by the variation in risk factors for uterine cancer or health care factors influencing the diagnosis of the disease [8]. The increasing rates in the Gharbiah province of Egypt over the study period could be related to both risk factors and health systems factors. It is unlikely that improvement in the population-based registries has been a factor, since these have maintained consistently high standards verified by peer review [17, 18].

Early age of menarche, late age of menopause, nulliparity, obesity, and diabetes mellitus are well-documented risk factors for uterine cancer in different countries.

Early age of menarche and late age of menopause are two risk factors related to increased risk for uterine cancer [19–23]. Average age of menarche in high- and low-SES girls in Egypt was reported in 1978 as 12.59 years and 13.89 years, for the 2 groups, respectively [24]. A more recent study showed that the mean age at menarche in Egypt declined to 12.49 years in the period of 2012–2013 [25]. Age of menopause has increased in Egypt over time. In 1986, a study from the Sharkia province in Egypt showed an average age of menopause of 45.2 years [26], and another study from Alexandria, Egypt, showed an average age of menopause of 45.8 years [27]. Studies from the rural province of Menoufia showed a lower average age of menopause of 43.1 years [28]. More recent studies showed a higher average age of menopause of 46.7 years and 50.84 years in Alexandria and Assiut, respectively [29–31].

Regarding the relationship between parity and uterine cancer, low parity has been considered a risk factor while high parity was considered a protective factor for the disease [32, 33]. The fertility rate among Egyptian women (15–49 years) was 3.5 births per woman in 2014. [34]. Although the fertility rate declined from 5.3 to 3.5 births per woman during the period of 1980 to 2014, the rate is still higher than that of developed countries. For example, the fertility rates in the US, France, and the UK were reported as 1.9, 2.0, and 1.9, respectively, in 2011–2015 [35].

Obesity is considered an important risk factor for endometrial cancer for both pre- and postmenopausal women. [36–41]. About 39% of the risk of endometrial cancer can be attributed to overweight and obesity [42]. Egypt has been considered one of the countries with the highest overweight and obesity rates [43]. The Egyptian Demographic and Health Survey (EDHS) showed an increase in the proportion of obese women from 40.85 to 48.1% during the period from 2000 to 2014 [34, 44–46]. According to the WHO estimate for 2010–2014, prevalence of obesity (BMI ≥ 30) among Egyptian females aged 18+ years was 37.5% (95% CI 30.5–44.9) [43]. A hospital-based study from Alexandria, Egypt, showed abdominal obesity as a significant risk factor for endometrial cancer (OR = 13.58, 95% CI (4.0–46.6)) [47]. Moreover, diabetes mellitus is a well-known risk factor [48–50]. The prevalence of diabetes mellitus among Egyptian women increased from 8% in 1980 to 18.2% in 2014 [51]. Therefore, the increase in some uterine risk factors over the past decades could explain the increasing incidence of uterine cancer in our study.

Egypt is one of the middle-income countries that have been undergoing epidemiologic and nutritional transitions [52–54]. For example, the prevalence of overweight and obesity was 28.2% and 7.6%, respectively, among Egyptian school girls of mean age of 13.2 during the period of 2006–2010 [40]. If increasing trends of overweight and obesity continue among Egyptian women, endometrial cancer might be expected to continue to increase in the future.

Although variations in risk factors may explain difference in incidence of uterine cancer between populations, protective factors may also play a role in variation in rates between countries. For instance, smoking is another risk factor that has been associated with low rates of uterine cancer in postmenopausal women [55–57] However, it should be noted that the smoking rate among Egyptian women is extremely low (0.5%) compared to the male population (37.7%) [58, 59]. However, these studies did not address the possibility of exposure of women to passive smoking.

Oral contraceptive pills and injectable contraceptive methods are considered protective against endometrial cancer [60, 61] The estimated prevalence of using birth control pills of Egyptian women of reproductive age (15–49 years) was 16.6% in 1980 and declined to 9.5% in 2000 then increased again to 16% in 2014 [62]. However, there has been a constant increase in the use of injectable contraceptive methods from 0.1% in 1988 to 8.5% in 2014 [34]. These rates of usage are quite low compared to rates in developed countries that reach 40% [62]. In addition, the increase in physical activity was found protective against uterine cancer [63–65]. About 57% of women living in rural areas in our study region were engaged in daily activities and work in extensive farm-related physical work, which demanded a considerable amount of physical activity [10]. Moreover, the different exposures to environmental factors may affect uterine cancer incidence. For instance, exposure to xenoestrogens as a component of environmental estrogen may increase uterine cancer rates [12].

About 36% of the patients included in our registry were in the age group of 50–59 years, and the age-specific rate was the highest (22.07 per 100,000) among the age group of 60 to 69 years. This result does not differ from uterine cancer data worldwide [66, 67]. The decrease in hormonal imbalance for both exogenous and endogenous hormones may explain the decline in uterine cancer incidence among elderly women [68]. Use of hormonal replacement therapy (HRT) by menopausal women is linked to increasing rates of uterine cancer [32, 69, 70]. However, HRT is rarely used in Egypt and is not known to most women in Egypt [30, 71].

Egypt has a wide-spread system of primary health care, and access to medical care in primary care, hospitals, and private clinics is not a major barrier to receiving medical care [10]. Also, availability of resources for imaging and pathologic diagnosis of cancer has improved significantly in Egypt [17, 18]. Whether these resources are available to physicians or underutilized for diagnosis of uterine cancer in this population is unknown.

Regarding the histopathologic types of uterine cancer, the majority of all types in this study were carcinomas and adenocarcinomas. The results of this study are in agreement with the classic presentation of adenocarcinoma among uterine cancer patients elsewhere. For example, adenocarcinomas comprise over 90% of all uterine cancers in the US [72]. However, in Tunisia, one of the North African countries, adenocarcinomas constituted about 63% of uterine cancers [73].

Most uterine cancers in this study were among postmenopausal women. This finding is consistent with studies from the US, where more than 90% of uterine cancer patients are postmenopausal [66].

The study has several strengths. The study builds on a track record of a reliable population-based cancer registry with validated data. The relatively long period of the study (12 years) adds to the strengths of the study. The coverage of medical care and accessibility to health care facilities in Egypt minimizes the chance of missed cases that had no access to medical care. Whether or not physicians histopathologically confirm the diagnosis of all uterine cancers could be a factor in underestimating uterine cancer in this population. Also, because of the low incidence of uterine cancer in this population, the relatively small sample size of this study might be a limitation.

In summary, while the uterine cancer incidence rate is low, but increasing over time in the Gharbiah province of Egypt compared to other countries, the postmenopausal Egyptian women comprised the vast majority of uterine cancer as in other countries. Future studies should focus on elucidating the impact of epidemiologic, demographic, and nutritional transitions on the future of uterine cancer in this population. Future studies should also investigate if uterine cancer is underestimated in this population, although this is unlikely due to population-wide accessibility to medical care in Egypt. Finally, research should explore if the increasing environmental pollution and environmental estrogens may have an impact on regional distribution of the disease in rural and urban regions of this population.

## Figures and Tables

**Table 1:** Demographic and clinical characteristics of 660 uterine cancer patients in the Gharbiah province between 1999 and 2010.

Variables	Frequency	Percent	95% CI	
			LL	UL
Menopausal status				
Premenopause	115	17.4	14.5	20.3
Postmenopause	545	82.6	79.7	85.5

Residential status				
Urban	355	53.8	50.0	57.6
Rural	305	46.2	42.4	50.0

Periods				
1999–2002	154	23.3	20.1	26.6
2003–2006	227	34.4	30.8	38.0
2007–2010	279	42.3	38.5	46.1

Age groups				
<40	23	5.0	3.3	6.7
40–49	82	12.4	9.9	14.9
50–59	237	35.9	32.2	39.6
60–69	217	32.9	29.3	36.5
≥70	91	13.8	11.2	16.4

Parity^(*n*=351)^				
Nulliparous	171	48.7	43.5	54.0
Low parity	21	6.0	3.5	8.5
High parity	159	45.3	40.1	50.5

Employment^(*n*=418)^				
Employed	48	11.5	8.4	14.6
Housewife	370	88.5	85.4	91.6

Basis of diagnosis				
Death certificate only	30	4.5	3.0	6.1
Histology of primary	614	93.0	91.1	95.0

Cancer topography				
Others	16	2.4	1.2	3.6
Corpus uteri cancer	599	90.9	88.7	93.1
NOS cancer	60	9.1	6.9	11.3

Tumor grades				
Well differentiated	94	14.2	11.6	16.9
Moderately differentiated	300	45.5	41.6	49.3
Poorly differentiated and anaplastic	110	16.7	13.8	19.5
Unspecified	156	23.6	20.4	26.9

Tumor stage^(*n*=560)^				
Localized	334	59.6	55.6	63.7
Regional	19	3.4	1.9	4.9
Distant	72	12.9	10.1	15.6
Unstaged	135	24.1	20.6	27.7

Cancer Histology^(*n*=656)^				
Adenocarcinoma	447	68.1	64.6	71.7
Other carcinoma	78	11.9	9.4	14.4
Sarcoma	72	11.0	8.6	13.4
Other unspecified malignant neoplasm	59	9.0	6.8	11.2

Treatment^(*n*=516)^				
No treatment given	120	23.3	19.6	26.9
Surgery alone	246	47.7	43.4	52.0
Surgery + adjuvant RT	98	19.0	15.6	22.4
Surgery + adjuvant RT and or adjuvant CT	52	10.1	7.5	12.7

**Table 2:** Age-specific incidence rate (per 100,000) of 660 uterine cancer diagnosed in the Gharbiah population of 1999–2010.

Age at diagnosis	Number of cases	Age-specific incidence rate/100,000	95% confidence interval	
			Lower limit	Upper limit
<40	33	0.19	0.13	0.26
40–49	82	2.94	2.35	3.63
50–59	237	12.52	11.00	14.19
60–69	217	22.07	19.28	25.16
≥70	91	16.65	13.49	20.34
Total	660	2.78	2.58	3.00

**Table 3:** The crude rate of uterine cancer in Gharbiah province for pre- and postmenopausal women with rate ratio (total *n*=660), 1999–2010.

Status	1999–2002			2003–2006				2007–2010			
	Crude rate	95%CI		Crude rate	95% CI	Crude rate ratio	95% CI	Crude rate	95% CI	Crude rate ratio	95% CI
Premenopausal	0.44	0.3–0.6	Ref	0.5	0.4–0.7	1.2	0.74–1.95	0.7	0.52–0.93	1.6	1.04–2.57
Postmenopause	10.87	8.96–12.78	Ref	16.7	14.37–19.12	1.5	1.23–1.95	20.2	17.55–22.77	1.9	1.49–2.31
Total	1.95	1.64–2.25	Ref	2.9	2.50–3.24	1.5	1.20–1.81	3.5	3.11–3.94	1.8	1.49–2.21

**Table 4:** The association of clinical characteristics and menopausal status of the uterine cancer cases (*n*=660) in the Gharbiah province, 2010.

	Menopause status				Pearson/exact chi-square
	Pre	Post	Pre	Post	
	*N*	*N*	Col (%)	Col (%)	
Cancer site					
Missing	0	1	—	—	<0.001
Corpus uteri cancer	95	504	83	93	
NOS cancer	20	40	17	7	

Basis of diagnosis					
Death certificate only	10	20	9	4	
Histology of primary	101	513	88	94	
Others	4	12	3	2	

Histology					
Missing	2	2	—	—	<0.001
Adenocarcinoma	57	390	50	72	
Other carcinoma	12	66	11	12	
Sarcoma	23	49	20	9	

Employment					
Missing	38	204	—	—	0.041
Employed	14	34	18	10	
Housewife	63	307	82	90	

Parity					
Missing	52	257	—	—	0.539
Nulliparous	33	138	52	48	
Low parity	5	16	8	6	
High parity	25	134	40	47	

Residential status					0.702
Urban	60	295	52	54	
Rural	55	250	48	46	

Stage					
Missing	14	86	—	—	0.975
Localized	62	272	61	59	
Regional	3	16	3	4	
Distant	13	59	13	13	
Unstaged	23	112	23	24	

Tumor grade					
Well differentiated	11	83	10	15	<0.001
Moderately differentiated	44	256	38	47	
Poorly differentiated and anaplastic	13	97	11	18	
Unspecified	47	109	41	20	

Treatment					
Missing	32	112	—	—	0.04
No treatment given	14	106	17	25	
Surgery alone	37	209	45	48	
Surgery + adjuvant RT	17	81	21	19	
Surgery + adjuvant RT and or adjuvant CT	15	37	18	9	

**Table 5:** The association of histology types of cancer and different clinical characteristics (*n*=660) in the Gharbiah province, 2010.

	Histology types				
	Adenocarcinoma	Other carcinoma	Sarcoma	Other and unspecified malignant neoplasms	*p*
	*N*	*N*	*N*	*N*	
Tumor grade					
Well differentiated	83	2	9	0	
	19	3	13	0	
Moderately differentiated	262	14	22	1	<0.0001
	59	18	31	2	
Poorly differentiated and anaplastic	65	21	23	0	
	15	27	32	0	
Unspecified	37	41	18	58	
	8	53	25	98	

Cancer site					
Corpus uteri cancer	440	69	72	15	<0.0001
	98	90	100	25	
NOS cancer	7	8	0	44	
	2	10	0	75	

Stage					
Missing	72	9	17	2	
Localized	245	42	35	10	
	65	61	64	18	
Regional	13	2	3	1	
	3	3	5	2	<0.001
Distant	37	13	14	7	
	10	19	25	12	
Unstaged	80	12	3	39	
	21	17	5	68	

Treatment					
Missing	102	25	13	22	
No treatment given	74	13	6	26	
	21	25	10	70	
Surgery alone	166	23	37	7	
	48	43	63	19	<0.0001
Surgery + adjuvant RT	68	12	12	0	
	20	23	20	0	
Surgery + adjuvant RT and or adjuvant CT	37	5	4	4	
	11	9	7	11	

## Data Availability

The data used for this study are available upon request.
